# Supplementary material for: Rising atmospheric moisture escalates the future impact of atmospheric rivers in the Antarctic climate system
Source: Commun Earth Environ. 2025 May 14;6(1):369. doi: 10.1038/s43247-025-02333-x (PMC12078180; doi:10.1038/s43247-025-02333-x)
Supplement: Supplementary file 2 — Supplementary Information [file 43247_2025_2333_MOESM2_ESM.pdf]

Supplementary Figures

For "Rising atmospheric moisture escalates the future impact of atmospheric rivers in the Antarctic climate system" by Maclennan et al.

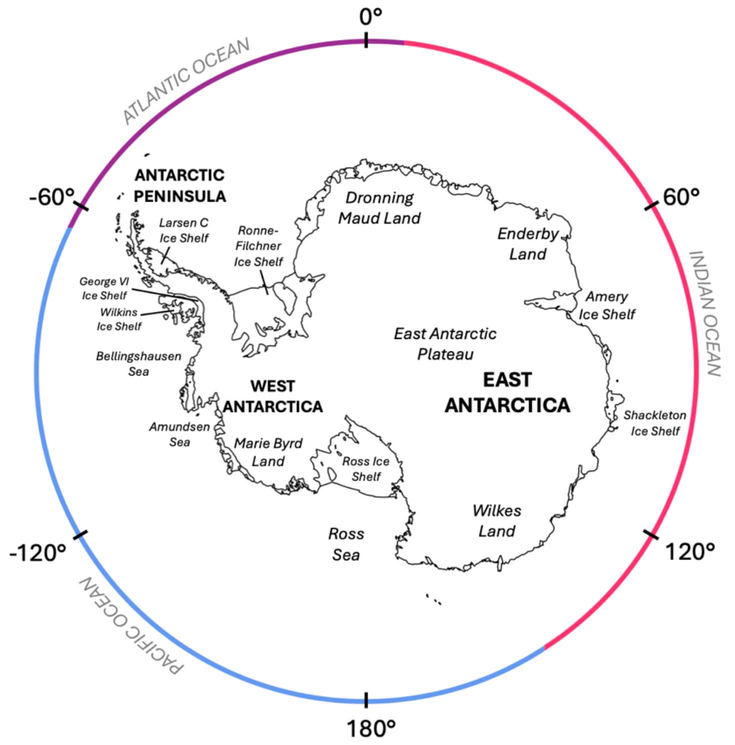

Figure S1: Map of Antarctica with regions mentioned in the manuscript labeled, along with adjacent ocean basins.

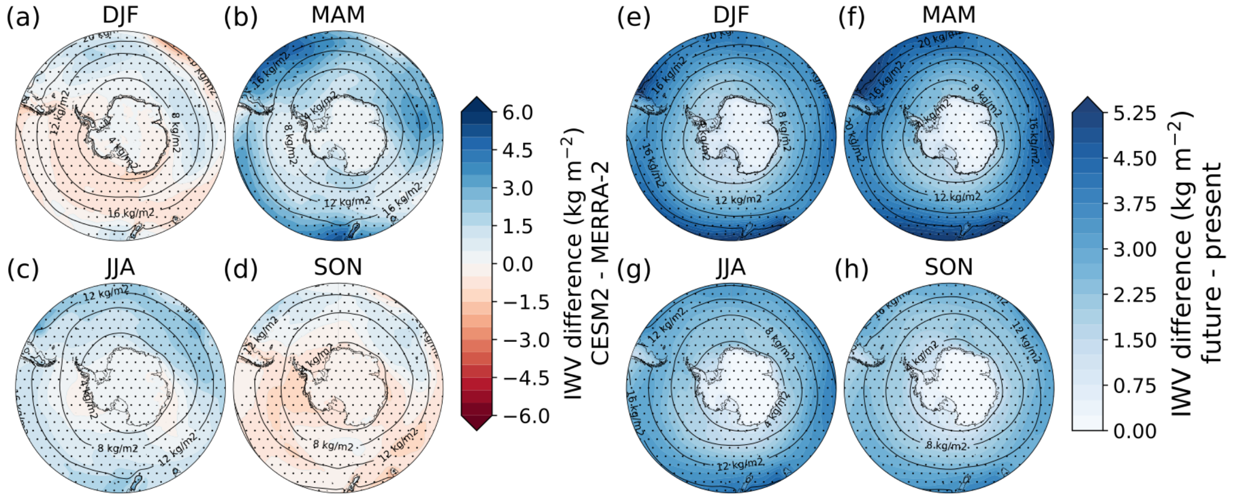

Figure S2: (a-d) CESM2 bias in integrated water vapor (IWV) relative to MERRA-2 over 1980 to 2014 in austral (a) summer (December-January-February), (b) fall (March-April-

May), (c) winter (June-July-August), and (d) spring (September-October-November). Black contours show the MERRA-2 IWV climatology over 1980 to 2014. Stippling indicates regions where the absolute value of the ensemble mean difference exceeds the standard deviation among ensemble members. (e-h) Change in IWV in CESM2 from 1980 to 2014 to 2066 to 2100 (future minus present), again for each season. Black contours show the CESM2 present-day climatology of IWV over 1980 to 2014. Stippling indicates regions of statistically significant difference based on a two-tailed z-statistic at the 95% confidence interval.

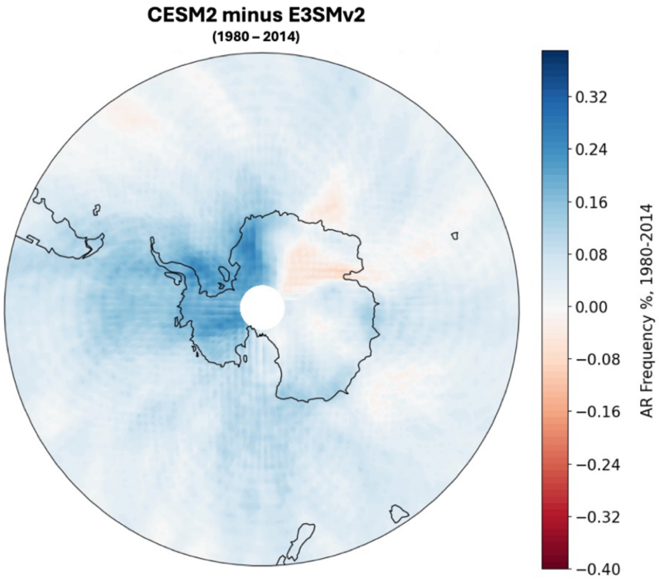

Figure S3: Difference in AR frequency between CESM2 LE and E3SMv2 LE for the historical simulations. For Antarctic ARs, the largest difference can be found in West Antarctica and western portions of Dronning Maud Land, where frequencies are larger in CESM2 compared to E3SMv2. The discrepancy between these two models can be tied to the mean sea level pressure representation between the two modeling frameworks, and their respective biases. Given these differences in the historical record, understanding the climate change response between these two models requires a deeper analysis and will be the subject of future studies.

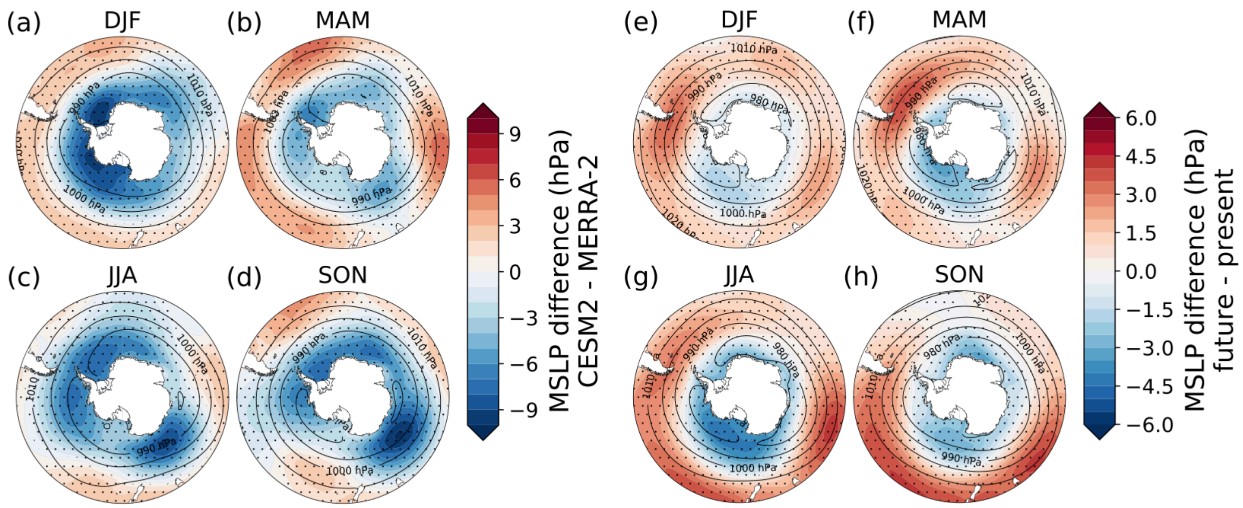

Figure S4: (a-d) CESM2 bias in mean sea level pressure (mslp) relative to MERRA-2 over 1980 to 2014 in austral (a) summer (December-January-February), (b) fall (March-April-May), (c) winter (June-July-August), and (d) spring (September-October-November). Black contours show the MERRA-2 mslp climatology over 1980 to 2014. Stippling indicates regions where the absolute value of the ensemble mean difference exceeds the standard deviation among ensemble members. (e-h) Change in mslp in CESM2 from 1980 to 2014 to 2066 to 2100 (future minus present), again for each season. Black contours show the CESM2 present-day climatology of mslp over 1980 to 2014. Stippling indicates regions of statistically significant difference based on a two-tailed z-statistic at the 95% confidence interval.

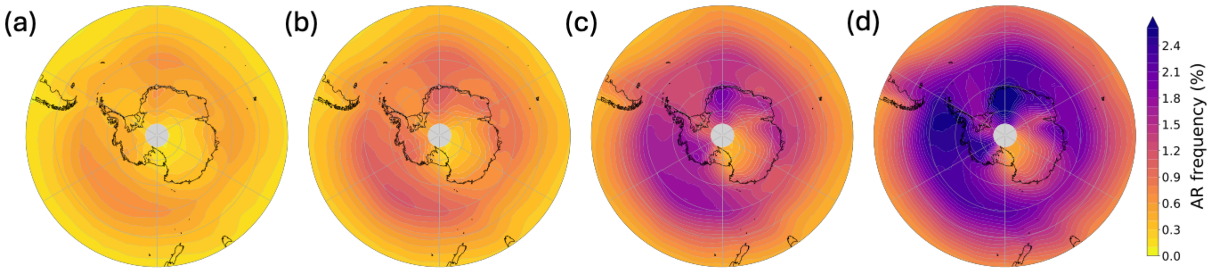

Figure S5: CESM2 ensemble mean annual AR frequency from 2066 to 2100 based on scaling for the increase in IWV with (a) the 90th percentile of the IWV ratio (most restrictive), (b) the mean IWV ratio, (c) the 10th percentile of the IWV ratio (less restrictive) and (d) no scaling, only using the present-day threshold for AR detection.

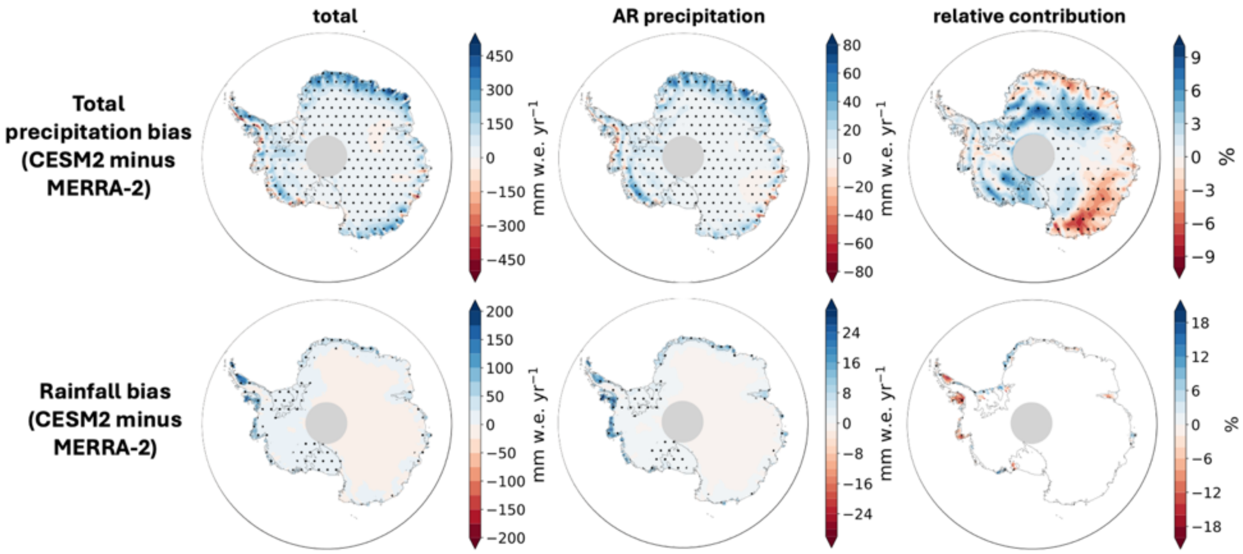

Figure S6: CESM2 precipitation bias relative to MERRA-2 from 1980 to 2014, with total precipitation (snowfall plus rainfall) in the top row and rainfall only in the bottom row. The first column shows the bias in all precipitation, the second column shows the bias in AR-attributed precipitation, and the third column shows the bias in the relative contribution of ARs to the total precipitation. Stippling indicates regions where the absolute value of the ensemble mean difference exceeds the standard deviation among ensemble members.

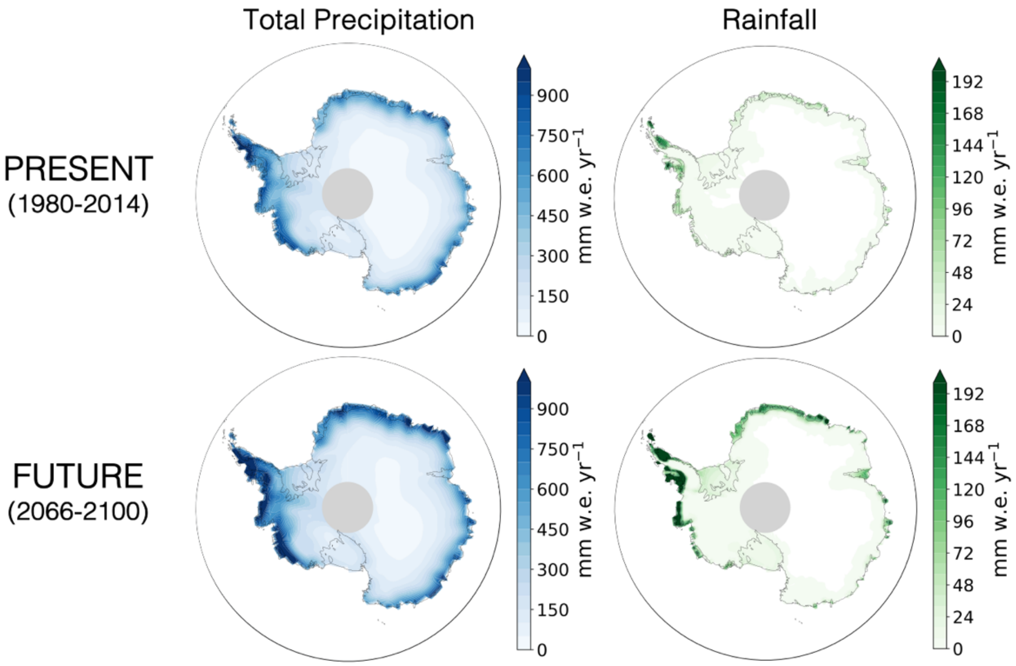

Figure S7: CESM2 annual mean total precipitation (left column) and total rainfall (right column) in the present climate (1980-2014, top row) and in the future climate (2066-2100, bottom row).

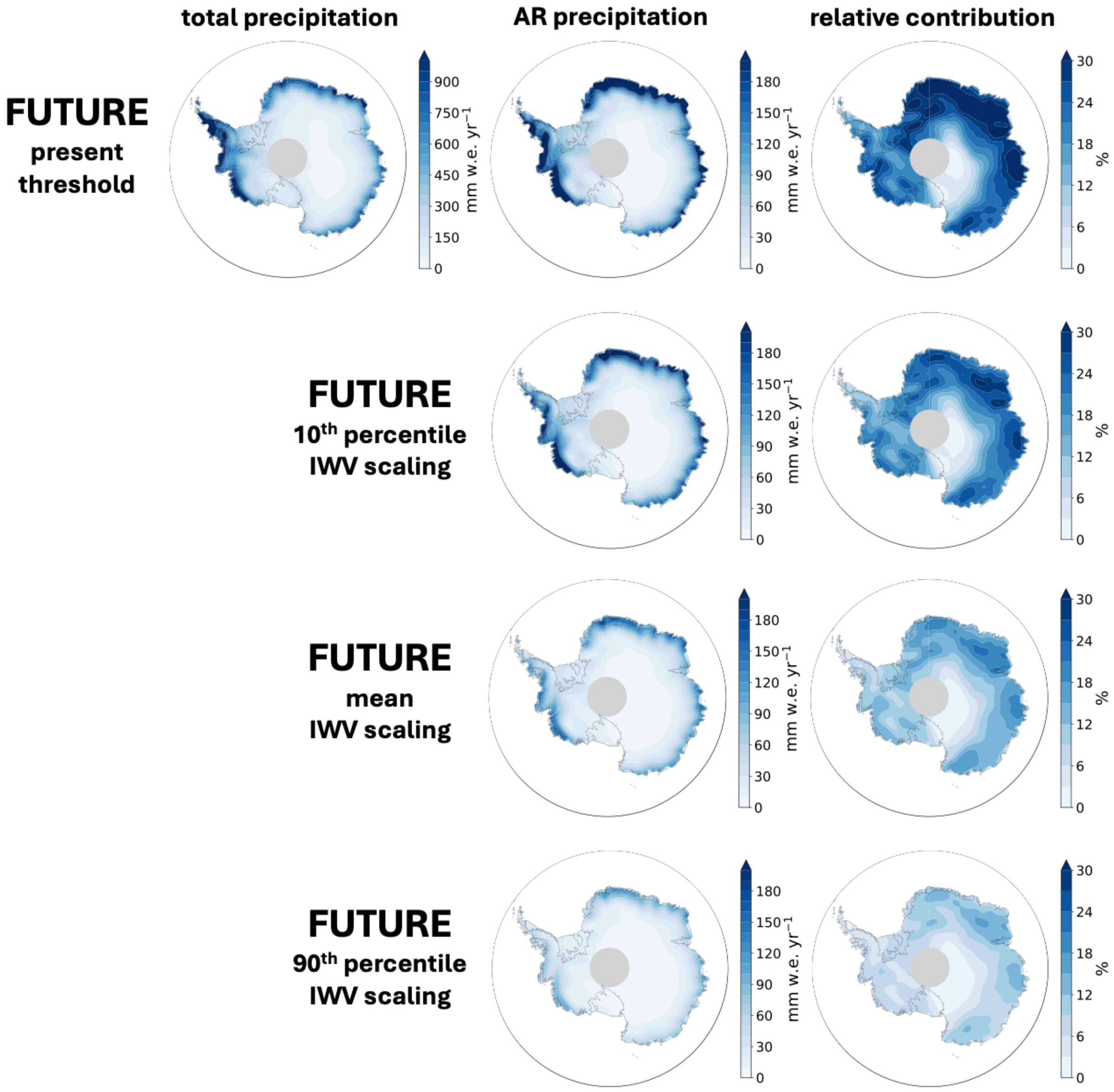

Figure S8: CESM2 future (2066-2100) annual mean precipitation (left), AR-attributed precipitation (center), and the AR contribution to total precipitation (right) under four different scaling scenarios for the Clausius-Clapeyron effect: (top row) the present day AR threshold (no scaling, least restrictive), (second row) scaling the present day threshold by the 10th percentile of the relative increase in IWV, (third row) scaling by the mean increase in IWV among ensemble members, and (fourth row) scaling the present day threshold by the 90th percentile of the relative increase in IWV (most restrictive).

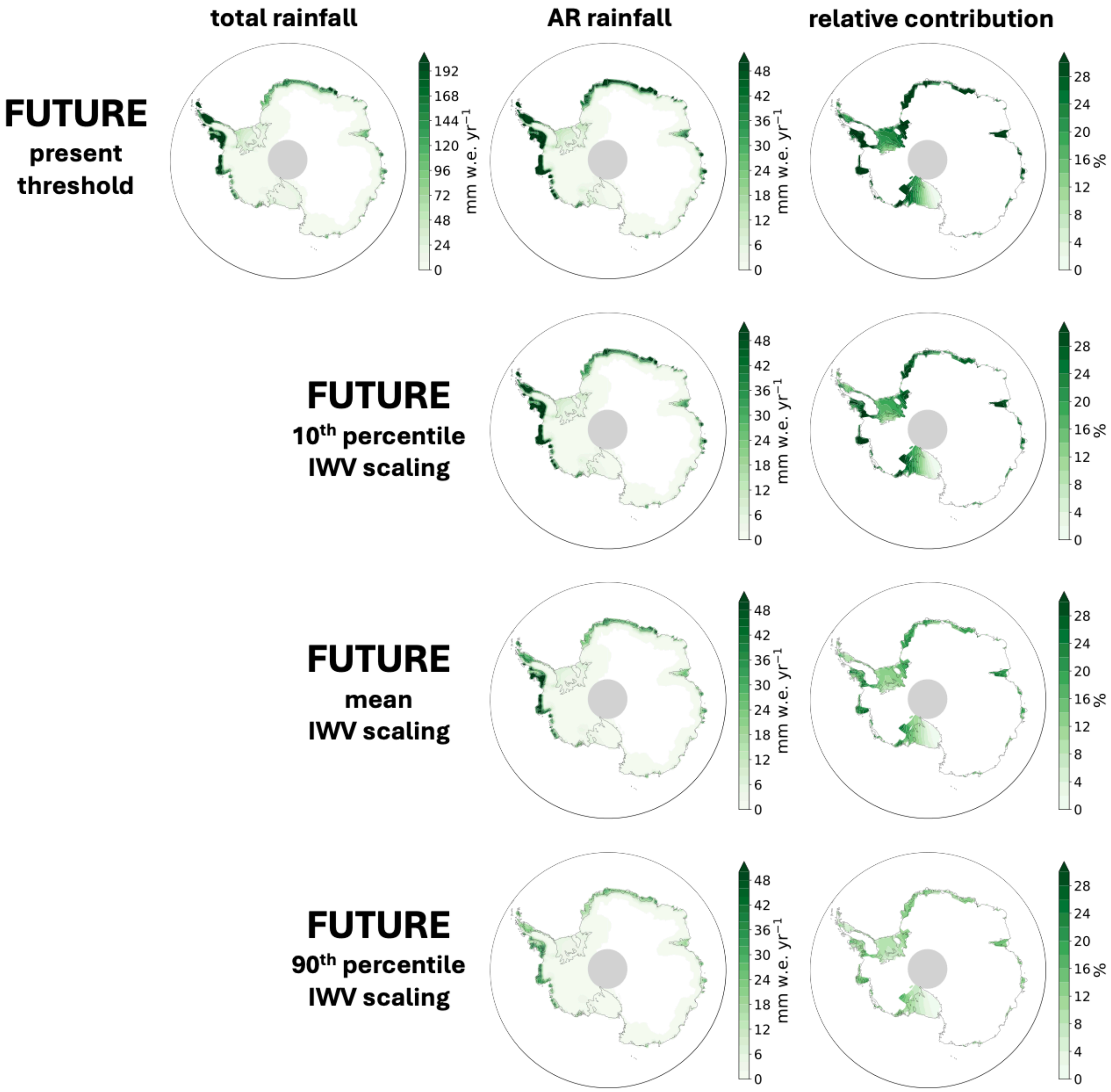

Figure S9: CESM2 future (2066-2100) annual mean rainfall (left), AR-attributed rainfall (center), and the AR contribution to total rainfall (right) under four different scaling scenarios for the Clausius-Clapeyron effect: (top row) the present day AR threshold (no scaling, least restrictive), (second row) scaling the present day threshold by the 10th percentile of the relative increase in IWV, (third row) scaling by the mean increase in IWV among ensemble members, and (fourth row) scaling the present day threshold by the 90th percentile of the relative increase in IWV (most restrictive).

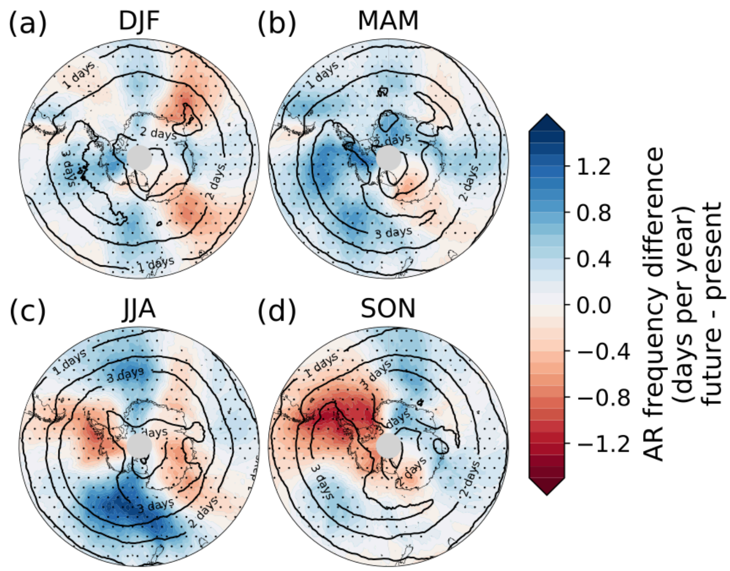

Figure S10: CESM2 ensemble mean change in AR frequency when scaling for the relative increase in IWV from 1980 to 2014 to 2066 to 2100 (future minus present) in austral (a) summer (December-January-February), (b) fall (March-April-May), (c) winter (June-July-August), and (d) spring (September-October-November). Black contours show the CESM2 AR frequency climatology over 1980 to 2014. Stippling indicates regions of statistically significant difference based on a two-tailed z-statistic at the 95% confidence interval.

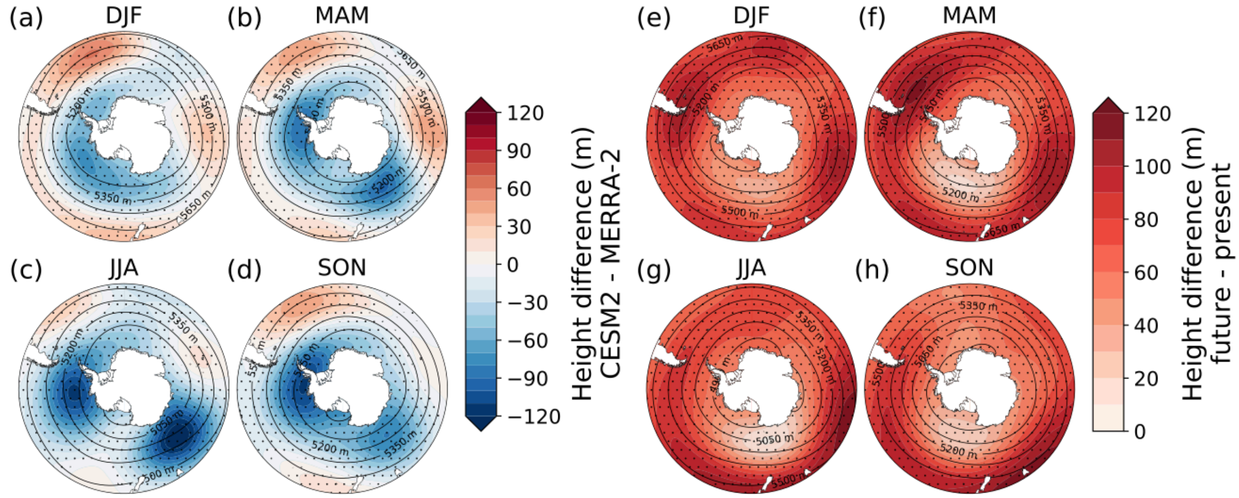

Figure S11: (a-d) CESM2 bias in 500 hPa geopotential height relative to MERRA-2 over 1980 to 2014 in austral (a) summer (December-January-February), (b) fall (March-April-May), (c) winter (June-July-August), and (d) spring (September-October-November). Black contours show the MERRA-2 500 hPa geopotential height climatology over 1980 to 2014. Stippling indicates regions where the absolute value of the ensemble mean difference exceeds the standard deviation among ensemble members. (e-h) Change in 500 hPa geopotential height in CESM2 from 1980 to 2014 to

2066 to 2100 (future minus present), again for each season. Black contours show the CESM2 present-day climatology of 500 hPa geopotential height over 1980 to 2014. Stippling indicates regions of statistically significant difference based on a two-tailed z-statistic at the 95% confidence interval.

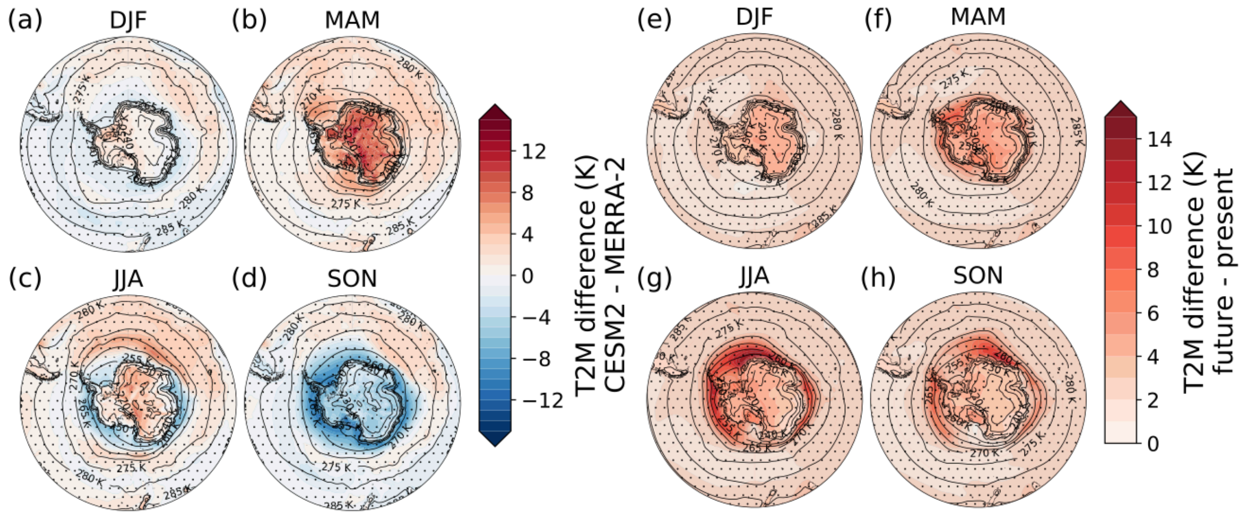

Figure S12: (a-d) CESM2 bias in 2m temperature relative to MERRA-2 over 1980 to 2014 in austral (a) summer (December-January-February), (b) fall (March-April-May), (c) winter (June-July-August), and (d) spring (September-October-November). Black contours show the MERRA-2 2m temperature climatology over 1980 to 2014. Stippling indicates regions where the absolute value of the ensemble mean difference exceeds the standard deviation among ensemble members. (e-h) Change in 2m temperature in CESM2 from 1980 to 2014 to 2066 to 2100 (future minus present), again for each season. Black contours show the CESM2 present-day climatology of 2m temperature over 1980 to 2014. Stippling indicates regions of statistically significant difference based on a two-tailed z-statistic at the 95% confidence interval.

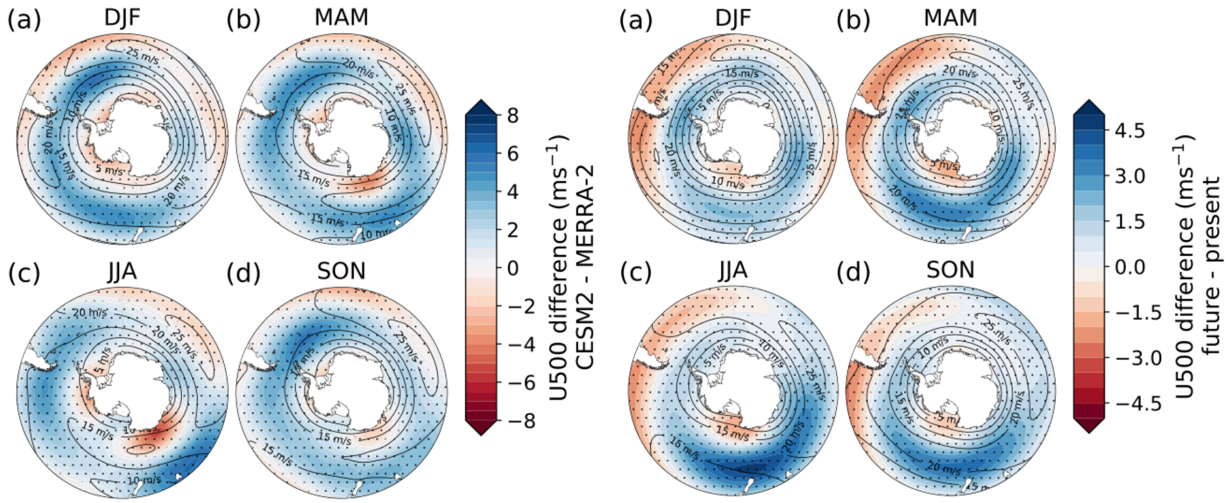

Figure S13: (a-d) CESM2 bias in 500 hPa zonal wind (U500) relative to MERRA-2 over 1980 to 2014 in austral (a) summer (December-January-February), (b) fall (March-April-May), (c) winter (June-July-August), and (d) spring (September-October-November). Black contours show the MERRA-2 500 hPa zonal wind climatology over 1980 to 2014. Stippling indicates regions where the absolute value of the ensemble mean difference exceeds the standard deviation among ensemble members. (e-h) Change in 500 hPa zonal wind in CESM2 from 1980 to 2014 to 2066 to 2100 (future minus present), again for each season. Black contours show the CESM2 present-day climatology of 500 hPa zonal wind over 1980 to 2014. Stippling indicates regions of statistically significant difference based on a two-tailed z-statistic at the 95% confidence interval.

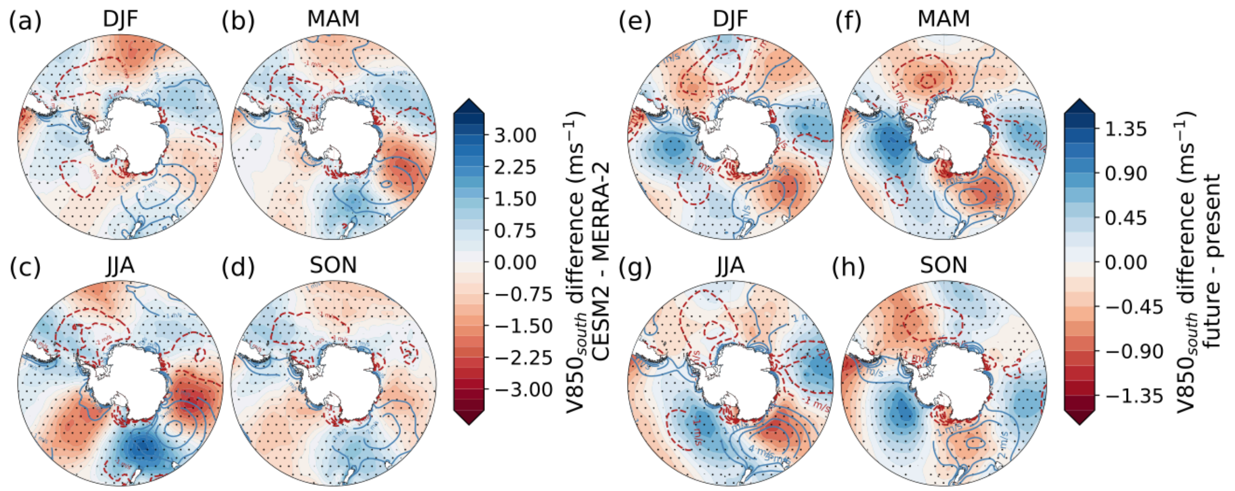

Figure S14: (a-d) CESM2 bias in 850 hPa poleward wind (V850) relative to MERRA-2 over 1980 to 2014 in austral (a) summer (December-January-February), (b) fall (March-April-May), (c) winter (June-July-August), and (d) spring (September-October-November). Contours show the MERRA-2 850 hPa poleward wind climatology over 1980 to 2014, with poleward winds in blue and equatorward winds in red. Stippling indicates regions where the absolute value of the ensemble mean difference exceeds

the standard deviation among ensemble members. (e-h) Change in 850 hPa poleward wind in CESM2 from 1980 to 2014 to 2066 to 2100 (future minus present), again for each season. Contours show the CESM2 present-day climatology of 850 hPa poleward wind over 1980 to 2014, with poleward winds in blue and equatorward winds in red. Stippling indicates regions of statistically significant difference based on a two-tailed z-statistic at the 95% confidence interval.

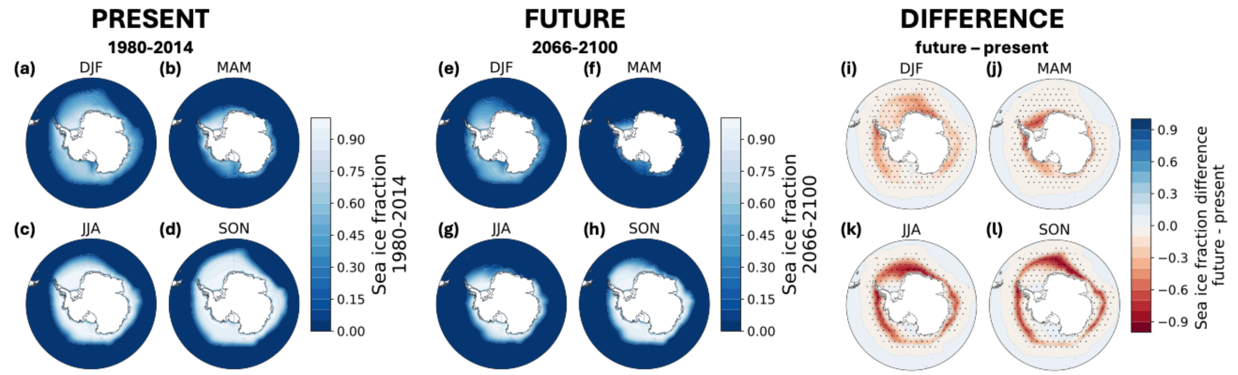

Figure S15: (a-d) CESM2 ensemble mean sea ice fraction from 1980 to 2014 in austral (a) summer (December-January-February), (b) fall (March-April-May), (c) winter (June-July-August), and (d) spring (September-October-November). (e-h) Ensemble mean sea ice fraction from 2066 to 2100 by season. (i-l) Ensemble mean difference in sea ice fraction between the future period (2066-2100) and the present (1980-2014) by season, with stippling indicating regions where the ensemble mean difference in sea ice fraction exceeds the standard deviation among ensemble members.

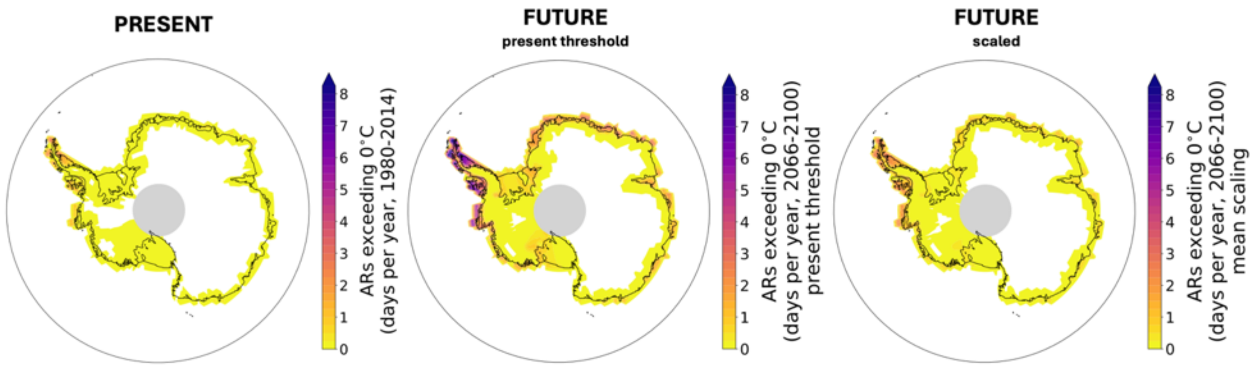

Figure S16: The annual frequency of ARs associated with surface air temperatures greater than or equal to 0° C in the present-day (1980-2014) and future climate (2066-2100) with the present-day threshold and the mean scaled threshold.
